# High-Compressive-Strength Silicon Carbide Ceramics with Enhanced Mechanical Performance

**DOI:** 10.3390/ma18153598

**Published:** 2025-07-31

**Authors:** Zijun Qian, Kang Li, Yabin Zhou, Hao Xu, Haiyan Qian, Yihua Huang

**Affiliations:** 1 College of Materials Science and Engineering, Nanjing Tech University, Puzhu South Road No. 30, Nanjing 211816, China; 202361103068@njtech.edu.cn (Z.Q.); 202461203211@njtech.edu.cn (K.L.); 15005178219@163.com (Y.Z.); xhzs@njtech.edu.cn (H.X.); 2 State Key Laboratory of High-Performance Ceramics and Superfine Microstructure, Shanghai Institute of Ceramics, Chinese Academy of Sciences, No. 588, HeShuo Road, Jiading District, Shanghai 201800, China

**Keywords:** reaction-bonded silicon carbide, compressive strength, particle gradation, liquid silicon infiltration, residual silicon

## Abstract

This study demonstrates the successful fabrication of high-performance reaction-bonded silicon carbide (RBSC) ceramics through an optimized liquid silicon infiltration (LSI) process employing multi-modal SiC particle gradation and nano-carbon black (0.6 µm) additives. By engineering porous preforms with hierarchical SiC distributions and tailored carbon sources, the resulting ceramics achieved a compressive strength of 2393 MPa and a flexural strength of 380 MPa, surpassing conventional RBSC systems. Microstructural analyses revealed homogeneous β-SiC formation and crack deflection mechanisms as key contributors to mechanical enhancement. Ultrafine SiC particles (0.5–2 µm) refined pore architectures and mediated capillary dynamics during infiltration, enabling nanoscale dispersion of residual silicon phases and minimizing interfacial defects. Compared to coarse-grained counterparts, the ultrafine SiC system exhibited a 23% increase in compressive strength, attributed to reduced sintering defects and enhanced load transfer efficiency. This work establishes a scalable strategy for designing RBSC ceramics for extreme mechanical environments, bridging material innovation with applications in high-stress structural components.

## 1. Introduction

Reaction-bonded silicon carbide (RBSC) ceramics have garnered significant attention as advanced structural materials due to their exceptional hardness, wear resistance, thermal stability, and near-net-shape manufacturability [1,2,3,4,5,6,7,8]. Extensive studies have explored the effects of SiC particle gradation and carbon source selection on flexural strength and thermal conductivity [2,9,10]. For instance, Liu et al. [2,11] demonstrated that hierarchical microstructures—combining coarse (60 µm) and fine (13 µm) SiC particles—enhanced thermal conductivity (up to 188 W/m K) but compromised bending strength due to reduced interparticle contact. Conversely, Wilhelm et al. [12] reported that submicron SiC powders (0.5–3 µm) improved bending strength (583 MPa) by refining residual silicon distribution, albeit at the expense of thermal performance. Carbon additives, such as nano-sized carbon black (1.3 µm) or phenolic resin-derived carbon, further influence porosity and reaction kinetics during liquid silicon infiltration (LSI). Our previous work [13] studied larger-grain-size particle reaction-bonded SiC and found that adding 8 wt% carbon black is good for mechanical properties. Zhang et al. [14] found that when carbon black and graphite were used as carbon sources, the residual silicon content was less than that when the carbon source was only graphite. The mechanical properties of reaction-sintered SiC ceramics are better when the carbon black content is 4% and the graphite content is 6%. Pan et al. [15] used nano-carbon black as an active carbon source, and excess carbon microspheres and diamond as inert carbon sources, respectively. It is found that the reaction rate of diamond is less than that of carbon microspheres, there is more residual silicon in the sample, the thermal conductivity is lower, but the hardness is higher. In addition, the most common way to toughen RBSC ceramics is to add second phases, such as whiskers, fibers, etc. Li et al. [16], through the introduction of SiC whiskers, demonstrated that the bulk density of the material is increased and the fracture toughness of the material is enhanced; Zhang et al. [17] used short fibers to interact with fiber debonding, crack deflection, and crack bridging, thus consuming crack energy and improving material toughness. These studies, however, predominantly focus on flexural properties, leaving the compressive behavior of RBSC—critical for load-bearing applications—largely unaddressed [18,19]. Pittari [18] introduced two types of RBSCs with high compressive strength (2.3 GPa), but they did not report their fabrication method. Wang et al. [20] reported a compressive strength of 2.8 GPa fabricated by the hot-pressing sintering method.

This knowledge gap is particularly problematic for extreme environments like deep-sea pressure hulls, where components must withstand multiaxial compressive stresses exceeding 100 MPa. While existing research on ceramics such as boron carbide highlights the sensitivity of compressive strength to defect distributions (e.g., graphitic inclusions and pores), analogous insights for RBSC remain scarce. For example, Hogan et al. [21] identified that graphitic disks (4.22 ± 2.54 µm) in hot-pressed boron carbide dominated compressive failure initiation, yet similar defect–property relationships in RBSC are unexplored. Moreover, conventional RBSC formulations often employ coarse SiC powders (6–60 µm) and micron-scale carbon sources, which may introduce stress-concentrating heterogeneities under compression. Venkatesan reported that the quasi-static compressive strength is much lower than the dynamic compressive strength for B_4_C ceramics [21]. DeVries et al. found that in relation to coarse-grained B_4_C, the ultrafine-grained material exhibited, on average, a 33% higher static compressive strength and 20% higher dynamic compressive strength [22].

Preliminary work by Paik et al. [1] demonstrated that bimodal SiC-C suspensions with 6.3 µm of SiC and 80 nm of carbon achieved flexural strengths of 310 MPa; here, we extend this approach to ultrafine systems targeting compressive performance [23]. This study proposes a novel RBSC design leveraging ultrafine SiC powders (0.5–2 µm) paired with nano-carbon black (<1 µm). The reduced particle size aims to enhance packing density, minimize critical flaw sizes, and promote homogeneous β-SiC formation during reactive sintering. Smaller SiC grains increase interfacial bonding areas, potentially improving load transfer under compression, while nanoscale carbon black optimizes pore size distribution to facilitate complete silicon infiltration.

## 2. Experimental Procedure

### 2.1. Preparation of Raw Materials

Raw materials include silicon carbide powder (D50 = 85 µm, D50 = 10 µm, D50 = 4 µm, D50 = 2 µm, D50 = 0.5 µm), graphite (D50 = 1.2 µm), carbon black (D50 = 0.6 µm), sodium carboxymethyl cellulose (CMC-Na), sulfonated melamine, sodium humate, deionized water, and silicon particles.

To fabricate the reaction-bonded silicon carbide (RBSC), two sets of silicon carbide (SiC) powders with different particle size distributions were utilized in Table 1. The primary set consisted of SiC powders with particle sizes of 2 µm (µm) and 0.5 µm, mixed in a weight ratio of 7:3 to achieve a multi-peak size distribution. This mixture was labeled as “CX-1” and “CX-2” with different solid loadings. The secondary set, serving as a comparison sample, was composed of SiC powders with particle sizes of 85 µm and 10 µm, 85 µm and 4 µm, also mixed in a weight ratio of 7:3, and labeled as “YX-1” and “YX-2” with different solid loadings. In order to distinguish mixtures of different carbon sources, we stipulate that YX-1, YX-2, CX-1, and CX-2 mentioned above are all mixtures with carbon black as the carbon source. The control groups with graphite as the carbon source were YX-1’, YX-2’, CX-1’, and CX-2’, respectively.

### 2.2. Slurry Preparation

All sets of SiC powders were mixed with a predetermined amount of carbon black (CB) or graphite as the carbon source. Sodium carboxymethyl cellulose (CMC-Na) was used as dispersant, sulfonated melamine as a water reducer, and sodium humate as a thickener. The CB or graphite content was fixed at 8 weight percent (wt%) for all samples to ensure consistency. The mixtures were then suspended in distilled water. SiC slurry with certain fluidity was prepared by using a high-speed vertical planetary mill and a vacuum defoaming mechanism. The speed of the ball mill was 300 r/min, and the milling time was 6 h.

### 2.3. Slip Casting and Green Body Formation

**Figure 1:**
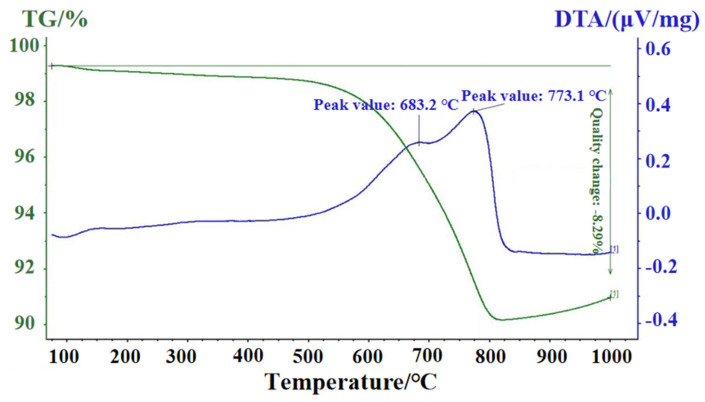
The TG-DTA curves of the C/SiC green body. The green line in the figure is the TG chart, and the blue line is the DTA chart.

The prepared slurry was poured into a 50 mm × 50 mm × 6 mm square mold and dried in an oven at 50 °C. The slurry was dried for 24 h and demolded to obtain the C/SiC green body. Finally, the green body was put into the high-temperature sintering furnace for debonding. The debonding temperature was raised to 900 °C at 2 °C/min in order to eliminate the polysaccharide organic matter, a small amount of internal bound water, and trace organic impurities, and obtain the porous SiC preform required for reactive infiltration. According to Figure 1, it can be observed that the binder began to decompose slowly from about 100 °C, and the thermal effect continued to increase until the peak temperature was 773.1 °C, the thermal effect change basically ended, and the main organic matter was decomposed. When the temperature was above 800 °C, the thermal effect inside the original C/SiC green body did not change significantly, but the quality increased slightly. It may be that the active carbon in the C/SiC green body reacts with gas or impurities to produce new substances at high temperatures, resulting in an increase in its quality.

### 2.4. Liquid Silicon Infiltration (LSI)

The porous preforms were placed into graphite crucibles, which were then coated with a boron nitride lubricating spray to prevent a reaction between the silicon and the crucible. Weigh an appropriate amount of silicon particles, with a mass ratio of 1:1.2 between silicon particles and prefabricated components, and use them to wrap prefabricated components. The crucibles containing the preforms were then heated to 1550 °C for 30 min in a vacuum environment to allow for liquid silicon infiltration. During this process, the liquid silicon infiltrated the pores of the preforms and reacted with the carbon source to form β-SiC, while the unreacted silicon remained as a residual silicon phase [24].

### 2.5. Sample Processing and Characterization

The rheological behavior of the slurry was characterized by a stress rheological instrument. The thixotropy history was eliminated by a pre-shearing treatment (100 s^−1^ for 60 s) before the test, and then the steady-state shear scanning (shear rate 1–100 s^−1^) was carried out at 25 °C to obtain the viscosity shear rate curve. The particle size distribution of SiC particles was measured by a laser particle size analyzer. The pore size distribution and size of the C/SiC porous preform precursor were analyzed by an automatic four-station specific surface aperture analyzer combined with the Mercury intrusion porosimetry (MIP, Poremaster 60, Anton Pacunta, McMurdrick, Norcross, GA, USA) analysis.

The polished surfaces and fracture surfaces of the samples were observed under an SEM (SEM, FEI, Hillsboro, OR, USA) to study their microstructures. The microstructures of the green bodies were also observed using a scanning electron microscope to ensure proper particle packing and pore formation. At the same time, an energy-dispersive spectrometer (EDS) was equipped to analyze the composition of the sample. Additionally, the relative density and residual silicon content of the samples were measured using Archimedes’ method and chemical corrosion, respectively.

The mechanical properties of these samples, including flexural strength and compressive strength, were measured using a universal testing machine (Instron-5566, Instron, Norwood, MA, USA). After LSI, the RBSC samples were processed into test strips of dimensions 3 × 4 × 36 mm for flexural strength. The sample for compressive strength testing is a cylinder with a diameter of 5 mm and a height of 12.5 mm, and the displacement rate of the crossbeam is 0.2 mm/min by the universal testing machine. By following this experimental procedure, the influence of different SiC particle size distributions on the microstructural and mechanical properties of RBSC could be systematically investigated and compared.

## 3. Results and Discussion

### 3.1. Selection of Carbon Sources

**Figure 2:**
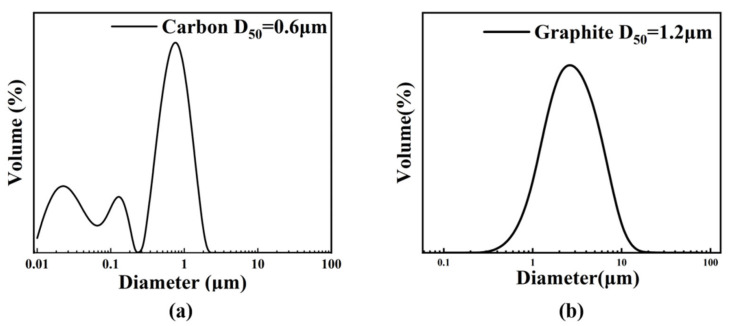
Particle size distribution of carbon source: (**a**) carbon; (**b**) graphite.

The particle sizes of the two types of carbon sources used in the experiment were analyzed by a laser diffraction particle size analyzer, as shown in Figure 2. The particle size distribution of carbon black is multi-modal, with a D50 of 0.6 µm and a D10 of 200 nm. The particle size distribution of graphite is a single peak, with a D50 of 1.2 µm. The chemical composition of graphite is carbon, and its crystal framework is a hexagonal layered structure, belonging to the hexagonal crystal system. It can also be used as a carbon source to react with silicon. The peak width of graphite is obviously larger than that of carbon black, the particle size is relatively divergent, and the carbon black is relatively concentrated near 0.6 µm. For the particle size requirements required by grading, the effect of multi-peak distribution is generally better than that of single-peak distribution. The micromorphology of carbon black further reveals the reasons for the formation of a variety of wide peaks.

**Figure 3:**
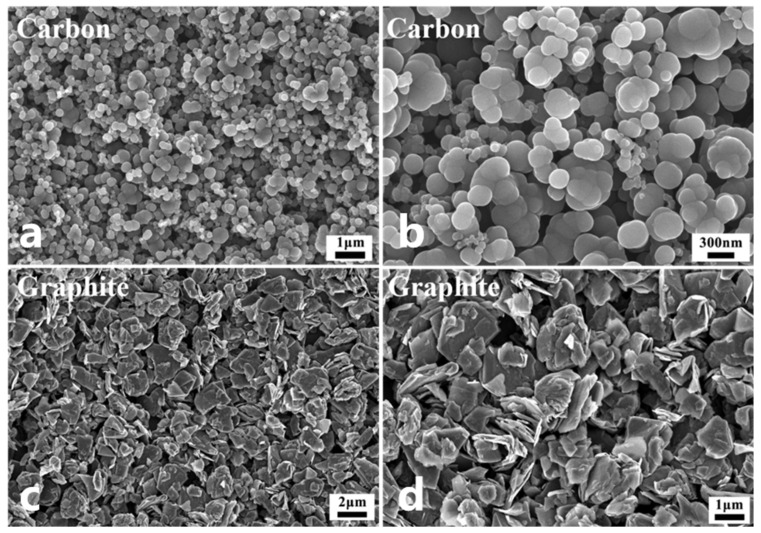
The SEM images of the carbon source: (**a**,**b**) carbon; (**c**,**d**) graphite.

The microscopic analysis of carbon black and graphite, as depicted in Figure 3 under varying magnifications, reveals stark contrasts in their structural and functional properties. Carbon black particles exhibit a microsphere-like morphology with significant size variations, where individual spherical particles (about 200 nm) agglomerate into larger clusters (about 1 µm) through electrostatic attraction, a phenomenon clearly visible in the images and directly responsible for the multi-modal particle size distribution observed in Figure 3a,b. Graphite displays a distinct layered structure composed of multiple thin sheets (about 2 µm) with complete cleavage in Figure 3c,d, endowing it with natural floatability but simultaneously introducing challenges in particle packing and accumulation due to its irregular flake-like geometry, which leads to reduced plasticity, inferior smoothness compared to the spherical carbon black, and a tendency for the flakes to stack and compress into dense blocks, resulting in lower fault tolerance during processing.

### 3.2. Properties of Slurry

**Figure 4:**
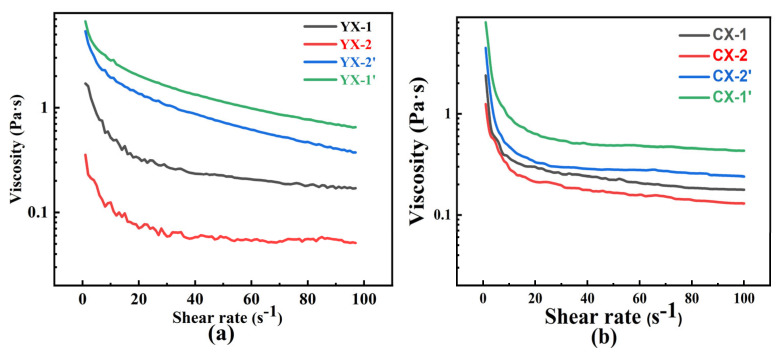
Rheological function of SiC slurries: (**a**) YX viscosity curve; (**b**) CX viscosity curve.

From Section 3.1, we know that the electrostatic agglomeration of carbon black particles brings challenges to slurry dispersion, but this phenomenon differs fundamentally from graphite’s inherent structural constraints. The lamellar morphology of graphite causes significant viscosity problems in the slurry system. As shown in Figure 4, the viscosity curves of the slurry are shown. Graphite’s layered, plate-like morphology induces pronounced viscosity complications in slurry systems. It can be seen from Figure 3 that the initial viscosity of graphite slurry exhibited an exceptionally high initial viscosity of 2 Pa s. This indicates significant interparticle friction and structural resistance. In contrast, carbon black’s isotropic spherical architecture facilitates particle mobility through minimized contact surfaces, thereby reducing viscous drag forces. This geometric advantage enables carbon black-containing slurries to achieve both enhanced fluid dynamics and greater particle loading capacity while maintaining processability.

It can be seen from Figure 4a that the viscosity of coarse SiC graded slurry (YX) increases with the increase in solid content from 43.5 vol% to 54.3 vol%. This phenomenon is related to the close packing effect of particles. The increase in the solid content of slurry leads to an increase in the number of particles per unit volume, the compression of the particle spacing, the significant increase in the collision probability, and the increase in the formation probability of local aggregates, thus increasing the friction resistance between particles and the fluid viscosity effect. The slurry with a maximum solid loading of 54.3 vol% exhibited smooth viscosity curves with minimal fluctuations. This indicates excellent slurry stability and suitability for slip casting, attributed to the optimized particle packing and reduced interparticle friction in the bimodal system. In contrast, Figure 4b illustrates the viscosity behavior of ultrafine SiC slurries (CX, 0.5–2 µm). Here, the viscosity decreased with lower solid loadings, but achieving high solid content proved challenging due to the inherent limitations of ultrafine powders. Specifically, ultrafine particles occupy a significant volume fraction in the slurry, leading to rapid viscosity escalation even at moderate solid loadings. For instance, slurries with ultrafine SiC typically reached practical viscosity limits at ~30 vol% solid loading, a threshold compatible with slip casting processes but substantially lower than that of coarse systems.

These results highlight a critical trade-off: smaller particle sizes inherently increase slurry viscosity due to heightened surface areas and interparticle interactions, yet ultrafine powders can still form stable, castable slurries at reduced solid loadings. This balance underscores the feasibility of employing ultrafine SiC (0.5–2 µm) for fabricating dense RBSC components, provided that solid loading is carefully optimized to ensure both processability and final microstructure integrity.

### 3.3. Blank Aperture Distribution

**Figure 5:**
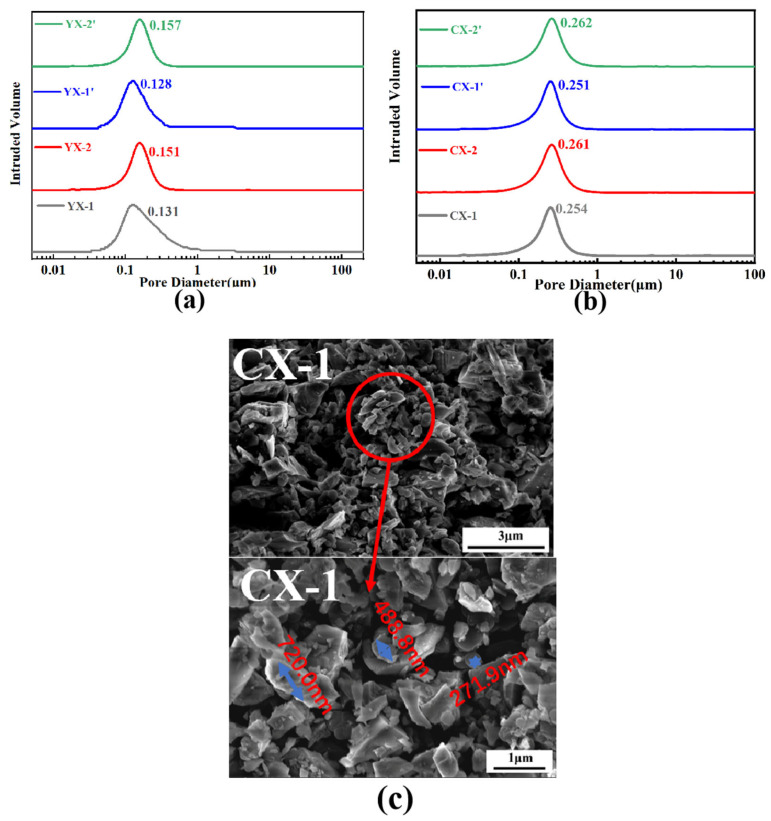
(**a**) The pore size distribution images for C/SiC preforms prepared with YX slurry. (**b**) The pore size distribution images for C/SiC preforms prepared with CX slurry. (**c**) SEM images for C/SiC preforms prepared with CX slurry.

Mercury intrusion porosimetry (MIP) analysis of the fabricated C/SiC preforms revealed distinct pore size distribution characteristics. Submicron C/SiC preforms exhibited narrow pore distributions, with carbon microspheres (<median pore size) effectively filling interparticle voids, as confirmed by SEM (Figure 5). These nano-carbon phases, dispersed between SiC grains, generated additional micropores while maintaining structural homogeneity. The bimodal system achieved optimal pore size distribution and microstructural integrity, demonstrating that two strategically sized fractions suffice to balance pore quantity, connectivity, and mechanical stability for subsequent silicon infiltration.

### 3.4. Microstructures of Si/SiC Ceramic

**Figure 6:**
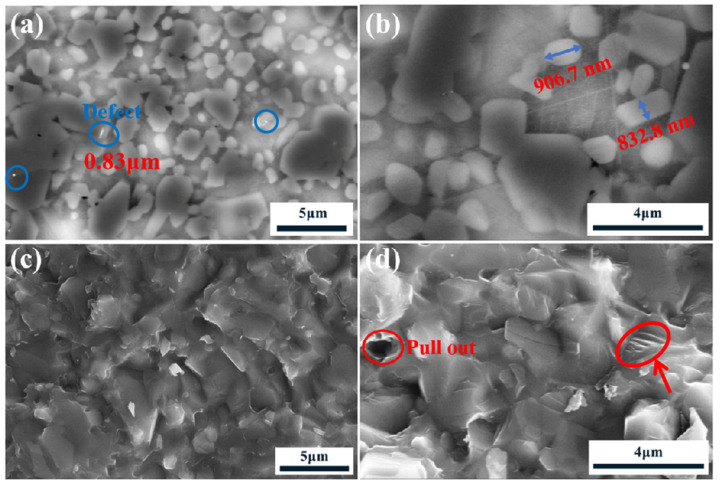
SEM images of Si/SiC samples prepared with CX-1 slurry: (**a**,**b**) the polished surface; (**c**,**d**) the fracture surface.

Figure 6 presents the microstructural evolution of Si/SiC ceramics fabricated using CX-1 ultrafine powders at varying magnifications. In Figure 6a, the microstructure is dominated by large SiC particles (light gray) interspersed with 0.5 µm black-gray pristine SiC particles, alongside a high population of secondary-phase SiC grains (small and gray-white). These secondary grains, formed via reactive infiltration of molten silicon with carbon black, exhibit a uniform distribution, suggesting efficient liquid-phase reaction kinetics and homogeneous nucleation.

Figure 6b reveals the elongated or columnar morphology of the secondary SiC grains, with lengths ranging between 200 and 500 nm. The anisotropic growth aligns with the β-SiC crystal habit, likely influenced by localized thermal gradients or carbon diffusion pathways during infiltration. Notably, the absence of equiaxed secondary grains implies preferential growth along specific crystallographic directions under the applied sintering conditions. According to Ovsienko’s report [25], these plate-like β-SiC could have formed by recrystallization of β-SiC plates or reaction of the Si melt with C produced via pyrolysis of the resin added as a binder during sample preparation.

Fracture surface analysis in Figure 6c demonstrates that intergranular fracture predominates, characterized by clean grain boundary separations with minimal transgranular cleavage. This fracture mode, though less frequent, reflects weaker grain boundaries relative to the bulk SiC matrix. In contrast, Figure 6d showcases rare instances of entire SiC grain pull-out, a phenomenon requiring substantial energy dissipation due to mechanical interlocking and frictional resistance. The sporadic occurrence of transgranular fracture, evidenced by uneven cleavage planes (arrows), correlates with localized stress fluctuations during bending, potentially linked to the ultrahigh strength (exceeding 2 GPa) of the ultrafine-derived Si/SiC. Hogan et al. [26] reported the effects of the processing-induced defect population on the dynamic compressive strength and failure of a hot-pressed boron carbide. In Figure 6a, the image software is used to analyze the defects. The results show that in this study, the defect size is below 1 µm, which is smaller than the 10 µm reported by Hogan.

**Figure 7:**
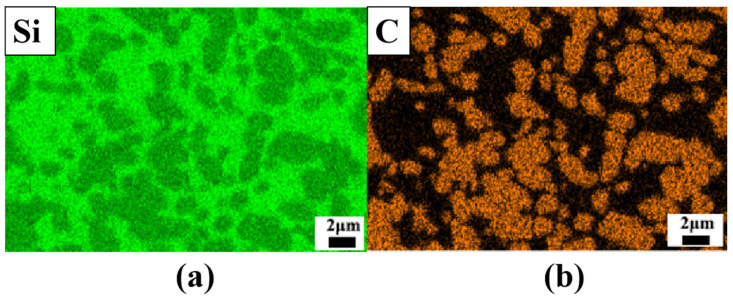
The energy spectrum face scan results of the Si/SiC sample prepared with the CX-1 sample. (**a**) Si; (**b**) C.

Figure 7 presents the energy-dispersive spectroscopy (EDS) elemental mapping of the CX-1 Si/SiC ceramic, where silicon (green) and carbon (orange) distributions are visualized. Due to the presence of Si in SiC crystals, the proportion of green appears to be relatively high. Notably, free silicon manifests as discrete, submicron-scale green clusters, uniformly dispersed at the interfaces between primary SiC grains and secondary β-SiC formed during reactive infiltration. These free silicon domains are spatially confined, with no evidence of large-scale aggregation or “silicon bands” exceeding the dimensions of the original SiC particles (typically <5 µm).

The homogeneous distribution of nanoscale free silicon originates from the optimized pore architecture and controlled capillary dynamics mediated by ultrafine SiC particulates (0.5–2 µm). Elemental carbon mapping (orange spectrum) confirms structural integrity of the SiC matrix with negligible residual carbon accumulation, demonstrating near-complete precursor conversion during liquid silicon infiltration. The elimination of macroscopic silicon agglomeration effectively circumvents stress localization and interface decohesion mechanisms, while the uniform dispersion of submicron silicon domains optimizes load transfer efficiency within the ceramic composite. This microstructural refinement directly contributes to the enhanced mechanical performance by reducing the dimensions and spatial uniformity of residual silicon phases. Such modifications reduce the probability of critical defect formation and establish tortuous crack propagation pathways through phase boundary engineering [12]. This study underscores the critical role of ultrafine SiC in tailoring free silicon morphology, offering a pathway to optimize RBSC ceramics for high-performance applications requiring both strength and microstructural reliability.

**Figure 8:**
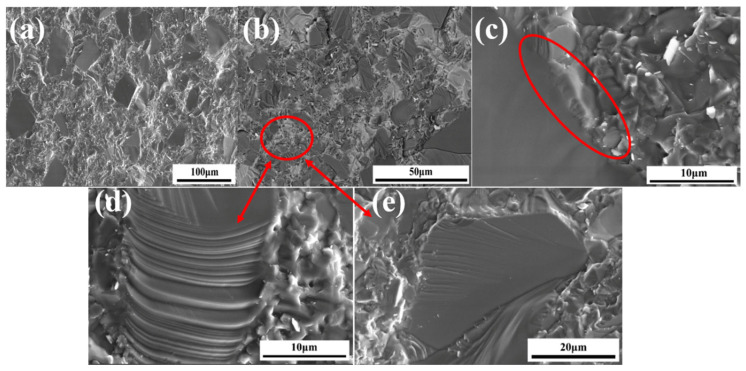
SEM images of Si/SiC samples prepared with YX-1 slurry: (**a**,**b**) the fracture surface; (**c**) free silicon, The light gray color in the circle represents free silicon, which accumulates between large grains and around the original silicon carbide grains; (**d**) small-scale cleavage surface; (**e**) small-scale cleavage surface.

The scanning electron microscope of YX-1 is shown in Figure 8. In Figure 8a, it can be clearly seen that the typical intergranular fracture occurred on the original large SiC particles, and many smooth surfaces with uneven light and shade are crystal fractures. In Figure 8b, it is observed that the cracks generated propagated along the grain boundary. It is captured in Figure 8c that Si is easy to gather between large particles or around the original SiC grain to form a precipitated phase of Si. Compared with the strong connection generated by β-SiC at the SiC grain boundary, this brittle Si band region destroys the stability of the grain boundary and reduces the mechanical properties of the material. Figure 8d,e show the grain planes of transgranular fracture after magnification. The steps between the mutually parallel cleavage planes at different heights in Figure 8d reflect the direction of fracture. The crack source is formed on the weak interface bonding the Si crystal at the grain boundary, and cleavage fractures are continuously generated along the direction of this weak interface. However, in Figure 8e, only a small range of river-like cleavage planes are generated on the grain surface, and the crack source is formed in the bright white impurity phase at the grain boundary and diffuses around, which is discontinuous and only locally expanded.

The above observations collectively demonstrate the critical role of ultrafine SiC powders in refining microstructure, enhancing interfacial cohesion, and mitigating defect-driven failure. The interplay between intergranular and transgranular fracture mechanisms further validates the exceptional mechanical integrity of the CX-1 system, positioning it as a promising candidate for high-stress structural applications.

Table 2 shows the content of free silicon in Si/Sic samples prepared by CX slurry and YX slurry. The proportion of residual silicon in reaction-sintered silicon carbide prepared by ultrafine powder is relatively higher, which also corresponds to the presence of more Si in Figure 7. Due to the small size and narrow pore size distribution of SiC, this kind of pore is not conducive to reducing the proportion of residual silicon as a whole. However, ultrafine SiC particles can reduce the accumulation of residual Si and can produce ultra-high-strength Si/SiC ceramic materials.

### 3.5. Mechanical Properties of Si/SiC Ceramic

**Figure 9:**
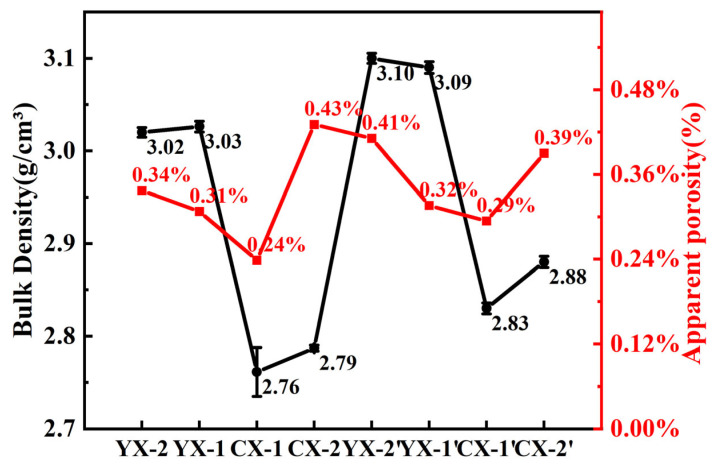
The bulk density and porosity of Si/SiC samples with different particle grading.

Comparing the density and porosity distribution of Si/SiC samples in Figure 9, it was found that after introducing large particles of SiC, the ceramic density of YX was significantly higher overall than that of Si/SiC ceramics prepared by ultrafine powder. Obviously, the characteristic of large particle size at the micro level can reduce the volume solid content occupied by the powder when the mass ratio is equal, directly increasing the density of the C/SiC preform and bringing the advantage of enhancing the volume density of Si/SiC ceramics. Due to the use of SiC particle size distribution, the filling degree in the pore distribution of C/SiC prefabricated parts is relatively high, and large pores are not generated, which makes it possible for liquid-phase silicon to remain between large SiC particles and avoid damaging the structural stability of the material. The pore connection method of the graded structure allows the silicon liquid to continuously and fully complete the infiltration reaction.

**Figure 10:**
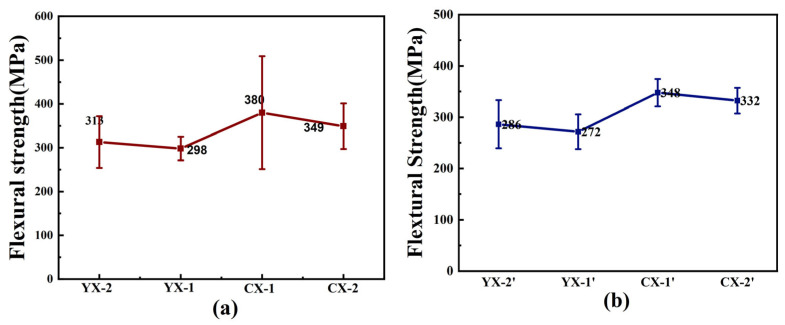
The flexural strength of Si/SiC samples prepared with YX and CX slurries. (**a**) flexural strength diagram of carbon black as carbon source sample; (**b**) flexural strength diagram of graphite as carbon source sample.

As depicted in Figure 10, the CX sample (fabricated with ultrafine SiC) exhibits higher flexural strength (exceeding 380 MPa) compared to the YX sample (conventional SiC), which achieved only ~300 MPa. This result is similar to Jang’s work [9], which used a bimodal SiC composition to reach a flexural strength of 475 MPa. This enhancement arises from the Hall–Petch effect, wherein the ultrafine SiC (0.5–2 µm) reduces the size of secondary-phase SiC and residual silicon to the nanoscale (10–500 nm). At this scale, dislocation glide dominates the fracture mechanism, requiring higher stress to propagate cracks through the refined, closely packed microstructure. However, when grain sizes are further reduced below ~50 nm (approaching the inverse Hall–Petch regime), grain rotation and boundary sliding become predominant, leading to strength degradation. Thus, the CX system represents an optimal balance between grain refinement and mechanical robustness. By comparing Figure 10a,b, it can be seen that the bending strength of the SiC material with graphite as a carbon source is low.

**Figure 11:**
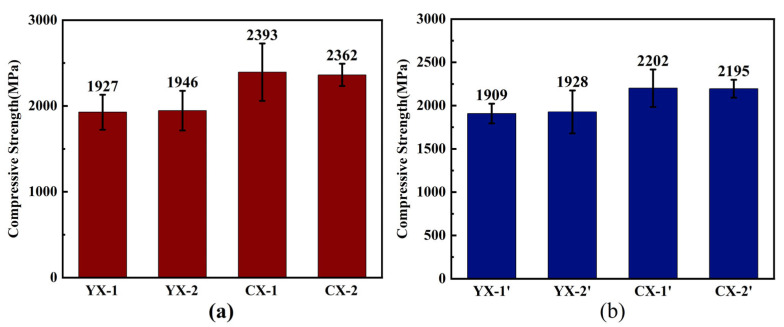
The compressive strength of Si/SiC samples prepared with YX and CX slurries. (**a**) compressive strength diagram of carbon black as carbon source sample; (**b**) compressive strength diagram of graphite as carbon source sample.

Figure 11 shows the compression properties of reaction-sintered silicon carbide materials prepared from YX and CX slurries. From the graph, it can be seen that the compressive strength of the CX material is high, and the maximum compressive strength of CX-1 is 2393 ± 372 MPa, while the compressive performance of the YX material is far lower than that of the CX material. Compared with Pittari’s report [18], the compressive strength of this work is comparable, but with a higher content of residual silicon. Hsu et al. reported that the tensile fracture occurred preferentially at the SiC/Si interface [27]. Although its grain size is smaller, the overall increase in compressive strength is limited. This value is obtained at a strain rate of 2.6 × 10^−4^/s by a universal testing machine. This value is smaller than the dynamic compressive strength (strain rate of 10^3^/s) reported by Pittari [18]. Compared with Wang’s work [20], this compressive strength is smaller, for hot-pressing sintering SiC has no residual silicon, which means there were fewer defects in the bulk. This is because the big-grain ceramics have bigger defects [28]. They thought that the presence of sintering defects like pores, which are due to some volatilization of the oxide secondary phases, would make the mechanical property decrease. In this study, the YX sample has bigger grains, which would lead to larger sintering defects. It can be seen from Figure 11 that the compression performance of silicon carbide material with graphite as the carbon source is also inferior to that of silicon carbide material with carbon black as the carbon source.

## 4. Conclusions

This study successfully demonstrates the fabrication of high-performance reaction-bonded silicon carbide (RBSC) ceramics via liquid silicon infiltration (LSI) combined with ultrafine SiC particle gradation (0.5–2 µm) and carbon black optimization. The resulting material exhibits exceptional compressive strength (2393 ± 372 MPa) and flexural strength (380 ± 20 MPa), surpassing conventional RBSC systems. Compared with the coarse-grained material, the compressive strength and flexural strength of the optimized RBSC increased by 23% and 20%, respectively, which was attributed to the minimization of sintering defects and the refinement of microstructure uniformity. Ultrafine SiC particles mediate capillary dynamics during infiltration, enabling uniform silicon distribution and minimizing residual carbon accumulation, as evidenced by elemental mapping and microstructural analyses. The elimination of macroscopic silicon agglomerations mitigates stress concentration risks, while submicron SiC domains enhance interfacial bonding and load transfer efficiency.

This work establishes a scalable strategy for tailoring RBSC ceramics to extreme mechanical environments, such as deep-sea pressure hulls or ballistic protection systems. Future efforts should focus on dynamic compressive behavior evaluation, interfacial phase engineering, and industrial-scale process optimization to bridge the gap between laboratory innovation and real-world applications. The findings underscore the critical role of particle gradation and carbon source selection in advancing structural ceramics for next-generation high-stress technologies.

## Figures and Tables

**Table 1:** The specific composition of the mixture of raw materials.

Samples	SiC Particle Size Distribution (D50)					Loading
	85 µm	10 µm	4 µm	2 µm	0.5 µm	
YX-1/YX-1’	70 wt%	30 wt%	0		0	54.3 vol%
YX-2/YX-2’	70 wt%		30 wt%			43.5 vol%
CX-1/CX-1’				70 wt%	30 wt%	32.1 vol%
CX-2/CX-2’				70 wt%	30 wt%	30.8 vol%

**Table 2:** The content of residual silicon for Si/SiC samples prepared with different particle grading.

Samples	YX-2	YX-1	CX-1	CX-2
Si/wt%	23.6	20.0	27.6	28.2

## Data Availability

The original contributions presented in this study are included in the article. Further inquiries can be directed to the corresponding authors.
